# From crisis to resilience: Catalysing epidemic and pandemic preparedness through National Public Health Institutes

**DOI:** 10.1016/j.ssmhs.2025.100165

**Published:** 2026-06

**Authors:** Thanitsara Rittiphairoj, Catherine Smallwood, Moa Herrgard, Wasiq Khan

**Affiliations:** a Department of International Health, Johns Hopkins Bloomberg School of Public Health, Johns Hopkins University, Baltimore, USA; b WHO Health Emergencies Programme, Regional Office for the Eastern Mediterranean, Amman, Jordan; c Chakri Naruebodindra Medical Institute, Faculty of Medicine Ramathibodi Hospital, Mahidol University, Bangkok, Thailand; d WHO Health Emergencies Programme, Berlin, Germany

**Keywords:** National Public Health Institutes, Emergency preparedness and response, Eastern Mediterranean Region, Pandemic, Health system resilience

## Abstract

The COVID-19 pandemic highlighted the critical role of National Public Health Institutes (NPHIs) in responding to global health emergencies. This commentary examined the contributions and challenges of NPHIs in four countries of the Eastern Mediterranean Region (EMR), including Somalia, Morocco, Pakistan, and Jordan, during the pandemic response. Drawing on country case studies, we highlighted shared achievements, unique contributions, and operational challenges. Key successes included strengthening public health surveillance systems, expanding laboratory testing capacities, establishing emergency operations centers, and advancing pandemic-related research. However, several systemic challenges impeded effective response, including insufficient funding, workforce shortages, weak health information systems, unclear leadership roles, lack of standardized operating procedures, and limited evidence use in policymaking. Based on these experiences, we proposed key strategies to strengthen NPHIs and improve pandemic preparedness. These included securing sustainable financing (e.g., contingency funds), coordinating donor support, investing in workforce training and simulations, improving peripheral laboratory infrastructure and data integration, developing standardized, multisectoral protocols led by NPHIs, strengthening international partnerships for surveillance and resource mobilization, and enhancing risk communication and community engagement. The paper emphasized the importance of sustained political commitment and long-term investment in NPHIs to build resilient health systems capable of effectively responding to future health threats.

## Introduction

The COVID-19 pandemic underscored the critical need for robust public health systems to respond effectively to health emergencies (DeSalvo et al., 2021). National Public Health Institutes (NPHIs) have been pivotal in this regard, coordinating responses and implementing strategies to mitigate the pandemic’s impacts (Zuber et al., 2022, Abou-Taleb et al., 2024). In the Eastern Mediterranean Region (EMR), countries with varying income levels and governance structures demonstrated the importance of NPHIs in pandemic preparedness and response (Khan et al., 2024). While prior literature broadly explores the role of NPHIs during COVID-19 (Zuber et al., 2022), there remains a gap in understanding how these institutions functioned in practice—including both their accomplishments and operational challenges—particularly in low- and middle-income country settings. This commentary aims to help fill that gap by presenting grounded examples from the region.

Our analysis drew on internal case studies developed by the World Health Organization Regional Office for the Eastern Mediterranean (WHO EMRO) (World Health Organization, 2024a, World Health Organization, 2024b, World Health Organization, 2024c, World Health Organization, 2024d). These case studies were informed by data from a 2023 survey of NPHIs, desk reviews, and consultations with key stakeholders, all of which documented national experiences during the COVID-19 response. We examined the cases of Somalia (World Health Organization, 2024a), Morocco (World Health Organization, 2024b), Pakistan (World Health Organization, 2024c), and Jordan (World Health Organization, 2024d)—countries selected based on the availability of materials and to reflect some variation in income level and institutional capacity within the region. By highlighting shared achievements, unique contributions, and key challenges faced by NPHIs during the pandemic response, we aim to offer practical insights to inform future efforts to strengthen public health institutions in epidemic and pandemic preparedness across the EMR.

## Achievements and challenges of National Public Health Institutions during the COVID-19 pandemic

During the countries’ COVID-19 responses, the NPHIs in Somalia, Morocco, Pakistan, and Jordan made significant contributions toward fulfilling the Essential Public Health Functions outlined by the International Association of National Public Health Institutes (IANPHI) and the World Health Organization (WHO) (World Health Organization, 2024e) (Table 1). One key area of system support was the delivery and enhancement of public health surveillance. The National Institute of Health (NIH) of Somalia, Institut National d′Hygiènes (INH) of Morocco, and NIH Pakistan played leading roles in enhancing laboratory and testing capacities (World Health Organization, 2024a, World Health Organization, 2024b, World Health Organization, 2024c). These efforts included establishing national testing networks, building new laboratories, deploying mobile testing units, and expanding laboratory information systems to support COVID-19 case surveillance and detection. These initiatives led to substantial increases in testing capacity—for example, Morocco’s PCR testing capability rose from 100 to 250,000 tests per day (World Health Organization, 2024b), while Pakistan’s increased from 30,000 to 280,000 (World Health Organization, 2024c).

**Table 1:** Contributions of National Public Health Institutes in Selected Countries to Essential Public Health Functions During COVID-19 Pandemic.

**Country**	**NPHI Name (Year Established)**	**2024 IANPHI/WHO Essential Public Health Function (**World Health Organization, 2024e**)**				
		**Public Health Surveillance**	**Public Health Emergency Management**	**Health Protection and Health Promotion**	**Public Health Workforce Development**	**Public Health Research, Evaluation and Knowledge**
**Somalia (**World Health Organization, 2024a**)**	NIH Somalia (2019)	• Enhanced molecular testing capacity by establishing national reference laboratory network	• Established multisectoral national incident management system • Established a temporary emergency operations center	• Disseminated health communication to the public using health bulletins, social and mass media	• Trained 44 epidemiologists through the Field Epidemiology Training Program (FETP)	
**Morocco (**World Health Organization, 2024b**)**	INH Morocco (1930)	• Strengthened real-time surveillance systems in coordination with the Ministry of Interior and its subordinate security services • Increased PCR testing capacity by establishing new sentinel sites and expanding existing laboratory information system	• Utilized existing incident management system designed to meet a range of emergency services • Collaborated with WHO, state and local health departments for emergency response		• Provided workforce training, both internally and externally, in areas related to detection of pathogens, biosafety and biosecurity, sequencing and biomolecular markers	• Launched genomic monitoring consortium for disease variants • Conducted research on phylogenetic trees to understand virus importation, transmission, and evolution • Collaborated with international partnerships in the EMR and EU on research and development
**Pakistan (**World Health Organization, 2024c**)**	NIH Pakistan (1965)	• Expanded testing capacity by establishing a national testing network and launching mobile vaccination outreach programs	• Established the National Command and Operations Center for national pandemic coordination	• Ran national and mobile campaigns to counter disease and vaccine misinformation	• Trained medical staff and lab technicians through existing FETP	• Conducted research on COVID-19 co-infections’ effects on respiratory diseases
**Jordan (**World Health Organization, 2024d**)**	Jordan CDC (2020)	• Developed a real-time case tracking system • Produced weekly reports on COVID-19 updates at the national, regional, and international levels.	• Actively participated in Ministry of Health’s Epidemiology Committee meetings, contributing to coordinated responses	• Disseminated health communication to the public using social and mass media	• Initiated workforce training through Rapid Response Teams and FETPs and supported capacity building of emergency operation centers	• Conducted extensive research, such as statistical models for COVID-19 prediction and studies on disease severity, spread, and mortality

EMR: Eastern Mediterranean Region; EU: European Union; FETP: Field Epidemiology Training Program; IANPHI: International Association of National Public Health Institutes; WHO: World Health Organization.

Surveillance data collection and sharing were also strengthened in Jordan, Morocco, and Somalia through the establishment or enhancement of event-based surveillance systems, enabling real-time monitoring of COVID-19 cases and hospitalizations (World Health Organization, 2024a, World Health Organization, 2024b, World Health Organization, 2024d). These systems supported timely, data-driven decision-making during rapidly evolving phases of the pandemic. In Jordan, the Center for Disease Control (CDC) played a critical role in communicating this data, providing weekly updates on COVID-19 to the Policy Council and relevant ministries, which helped guide national response efforts (World Health Organization, 2024d).

The second major area of contribution was public health emergency management. Emergency operations centers (EOCs) were established in Somalia and Pakistan to coordinate national pandemic responses (World Health Organization, 2024a, World Health Organization, 2024c), while INH Morocco leveraged its pre-existing emergency preparedness and response (EPR) mechanisms (World Health Organization, 2024b). In Somalia, NIH led the creation of the country’s first multisectoral incident management system, supported by a temporary public health EOC, to coordinate its COVID-19 response (World Health Organization, 2024a). Both NIH Pakistan and INH Morocco drew on established EPR systems (World Health Organization, 2024b, World Health Organization, 2024c); however, given Pakistan’s decentralized health governance structure, NIH also established a National Command and Operations Center to coordinate more effectively across provinces and federal entities (World Health Organization, 2024c). Lastly, NPHIs in lower-income countries, like NIH Somalia, INH Morocco, and NIH Pakistan, mentioned their close collaboration with WHO (World Health Organization, 2024a, World Health Organization, 2024b, World Health Organization, 2024c), which played a key role in strengthening health emergency management through technical guidance, surge support, and capacity-building during crises.

Public health research was another key domain of NPHI engagement. Jordan CDC stood out for generating critical insights into the virus spread through predictive modeling and statistical approaches to understand trends and estimate infection rates (World Health Organization, 2024d), while INH Morocco led the formulation of a national genomic monitoring consortium to study SARS-CoV-2 variants (World Health Organization, 2024b), contributing to both national surveillance and global research efforts.

In addition to these shared accomplishments, each country demonstrated unique strengths in its pandemic response. In health protection and promotion, NIH Pakistan led effective health promotion campaigns to counter misinformation and build vaccine confidence, achieving an 85 % vaccination coverage rate within approximately one year of receiving its first vaccine shipment (World Health Organization, 2024c). These efforts were supported by mobile vaccination units and targeted outreach strategies. In public health workforce development, NIH Somalia launched its first Field Epidemiology Training Program (FETP) shortly before the pandemic, which became instrumental in supporting the response (World Health Organization, 2024a). Meanwhile, NIH Pakistan scaled up its existing FETP, deploying trained personnel for case investigations, contact tracing, and surveillance activities (World Health Organization, 2024c).

However, the pandemic revealed systemic challenges in national health emergency management systems that could not be met within a short timeframe. One major issue was the lack of sustainable and well-coordinated funding for EPR (World Health Organization, 2024a, World Health Organization, 2024b, World Health Organization, 2024c, World Health Organization, 2024d). With insufficient domestic investment, many countries relied heavily on external funding during the pandemic (Barkia et al., 2021, African Development Bank Group, 2020, Asian Development Bank, 2024, International Monetary Fund, 2020). While donor support was instrumental in facilitating rapid response efforts, this reliance sometimes resulted in fragmented and short-term solutions, especially when funding streams were project-specific or time-limited (World Bank Group, 2022). This made it challenging to build cohesive and sustained EPR systems, leaving health systems vulnerable to future emergencies. Compounding these challenges were shortages of trained public health professionals, which placed further strain on already fragile systems, especially as demand for public health and clinical services surged (World Health Organization, 2024a, World Health Organization, 2024b, World Health Organization, 2024c, World Health Organization, 2024d). Weak health information management systems (HIMS) and barriers to data sharing further fragmented data collection and hindered outbreak monitoring (World Health Organization, 2024a).

In some countries, such as Jordan, the unclear designation of a lead response agency—or the lack of sufficient legal authority for the designated agency—undermined coordinated emergency planning (World Health Organization, 2024d). This leadership gap contributed to the absence of standardized operating procedures (SOPs) (World Health Organization, 2024d), which in turn hindered the generation of real-time public health intelligence. As a result, critical decision-making was delayed, impeding timely resource distribution and, in some cases, costing lives. Collectively, these systemic weaknesses undermined the use of evidence-based policymaking (Brownson et al., 2009)—a key global shortcoming (Organisation for Economic Co-operation and Development, 2020) that was evident in many EMR countries.

## Reflections on strengthening National Public Health Institutions and pandemic preparedness

Drawing from the experiences of the countries included in this commentary, several potential areas for strengthening EPR capacities emerged. While not intended as prescriptive recommendations, these reflections highlighted opportunities where future policy attention, investment, and research may be especially valuable:

1. Sustainable Financing: Long-term investment in EPR remains a foundational need (Kwon and Kim, 2022). Across the case studies, countries frequently faced fragmented or short-term funding (World Health Organization, 2024a, World Health Organization, 2024b, World Health Organization, 2024c, World Health Organization, 2024d), which undermined the continuity and effectiveness of preparedness efforts. Establishing contingency funds—dedicated national reserves earmarked for immediate use during public health emergencies—can help ensure timely resource mobilization when external funding is delayed or unavailable (World Health Organization, 2017). In parallel, coordinating and streamlining donor support through established national institutions such as NPHIs may reduce duplication, improve accountability, and enable more sustained and efficient allocation of resources aligned with national priorities (Mulemea et al., 2025).
2. Workforce Development: Expanding and retaining a skilled EPR workforce is essential (Sriram et al., 2024, Herstein et al., 2021). Many countries reported shortages of trained public health professionals, particularly in operationalizing rapid response systems and maintaining surge capacity (World Health Organization, 2024a, World Health Organization, 2024b, World Health Organization, 2024c, World Health Organization, 2024d). These challenges suggest that while theoretical knowledge may be present, practical skills—such as inter-agency coordination, field-based investigation, and real-time decision-making—often require reinforcement. Investments in FETPs and simulation exercises can help bridge these gaps by providing hands-on experience and fostering multisectoral coordination (Doble et al., 2023). These approaches can also help institutionalize a more practice-ready and resilient workforce, reducing reliance on ad hoc or external personnel during emergencies. Partnering with academic and training institutions can further enhance long-term workforce sustainability (Ruffolo et al., 2023).
3. Strengthened Infrastructure and Connected Data: Limited laboratory capacity and fragmented health information systems were recurring bottlenecks. Case study experiences showed that surveillance capacity improved with expanded lab infrastructure (World Health Organization, 2024a, World Health Organization, 2024b, World Health Organization, 2024c, World Health Organization, 2024d). However, siloed or incompatible HIMS often hindered data integration and timely decision-making (World Health Organization, 2024a). Targeted investment in regional lab infrastructure, interoperable HIMS adaptable to emergencies, and digital tools for real-time surveillance can enhance system resilience (Wolford et al., 2023, Ezenwaji et al., 2024). These improvements can boost diagnostic capacity, ease pressure on national or subnational labs, and expand access to underserved populations.
4. Standardised Protocols and Agile Updates: The absence or inconsistent application of SOPs was cited as a barrier to coordinated responses (World Health Organization, 2024d). Developing SOPs in advance, with input from multisectoral stakeholders, and aligning them with both global standards and local contexts can improve clarity and speed in emergency decision-making (World Health Organization, 2015). Given the rapidly evolving nature of health emergencies, it is equally important to establish mechanisms for regular review and timely update of protocols. NPHIs can lead and coordinate this process by convening technical committees that ensure guidance remains relevant and actionable throughout a response.
5. Leverage Partnerships: International and regional collaborations are crucial in enhancing NPHI capacity and overall preparedness (Brugnara et al., 2023). Case studies, particularly from lower-income countries, highlighted the value of cross-border and regional networks in mobilizing resources and coordinating surveillance activities (World Health Organization, 2024a, World Health Organization, 2024b, World Health Organization, 2024c). Such partnerships can also support joint training, resource sharing, and collaborative procurement. NPHIs are well-positioned to serve as conveners and facilitators of these partnerships, embedding them within national systems and enhancing resilience to future threats (Torjesen, 2020).
6. Risk Communication and Community Engagement: Limited involvement of NPHIs in health promotion and protection functions (as shown in Table 1) suggests a critical gap in preparedness. Effective and inclusive risk communication and community engagement are key to building public trust, combating misinformation, and encouraging adherence to health measures (Abdalla et al., 2021, Khan et al., 2022). Culturally tailored outreach, partnerships with civil society and community leaders, and training programs for community volunteers can strengthen social resilience and foster local ownership of preparedness activities. To ensure sustainability, community engagement should be integrated into local preparedness planning and not only activated during the response phase (World Health Organization, 2024f).

## The way forward

The COVID-19 pandemic exposed vulnerabilities in global health systems while highlighting the important role of NPHIs in crisis response. The experiences of Somalia, Morocco, Pakistan, and Jordan provided valuable insights into how NPHIs can drive emergency preparedness efforts, strengthen health systems, and foster resilience for future crises. By addressing funding gaps, workforce shortages, weak data infrastructure, fragmented partnerships and protocols, and limited involvement in risk communication and community engagement in EPR, countries can empower their NPHIs as central pillars of public health security.

While the pandemic created the need for urgent action to scale up health responses, it also drew attention and resources from the highest levels of government—creating a window for rapid innovation and change. As the COVID-19 emergency subsides, there remains an opportunity for countries to sustain these gains by rationalising and institutionalising them for long-term impact.

To ensure these gains are not lost, countries should prioritize embedding NPHIs within enduring national structures through clear legal mandates, sustainable financing, and integration into broader health system governance. Aligning these efforts with global momentum—such as WHO’s technical and strategic support (Khan et al., 2024)—can further enhance institutional capacity and peer learning. Investing in NPHIs as permanent, empowered institutions is not only critical for future pandemic preparedness, but also foundational to building resilient, responsive, and trusted public health systems.

## CRediT authorship contribution statement

**Thanitsara Rittiphairoj:** Writing – review & editing, Writing – original draft, Formal analysis, Conceptualization. **Catherine Smallwood:** Writing – review & editing. **Moa Herrgard:** Writing – review & editing. **Wasiq Khan:** Writing – review & editing, Conceptualization.

## Funding

The authors have not declared a specific grant for this research from any funding agency in the public, commercial or not-for-profit sectors.

## Declaration of Competing Interest

The authors declare that they have no known competing financial interests or personal relationships that could have appeared to influence the work reported in this paper.
